# There is a U shaped association between non high density lipoprotein cholesterol with overall and cardiovascular mortality in chronic kidney disease stage 3–5

**DOI:** 10.1038/s41598-020-69794-2

**Published:** 2020-07-29

**Authors:** Hsuan Chiu, Pei-Yu Wu, Jiun-Chi Huang, Hung-Pin Tu, Ming-Yen Lin, Szu-Chia Chen, Jer-Ming Chang

**Affiliations:** 1 Department of General Medicine, Kaohsiung Medical University Hospital, Kaohsiung, Taiwan; 2 Division of Nephrology, Department of Internal Medicine, Kaohsiung Medical University Hospital, Kaohsiung Medical University, Kaohsiung, Taiwan; 3 Department of Internal Medicine, Kaohsiung Municipal Siaogang Hospital, Kaohsiung Medical University, 482, Shan-Ming Rd., Hsiao-Kang Dist., Kaohsiung, 812 Taiwan, ROC; 4 Faculty of Medicine, College of Medicine, Kaohsiung Medical University, Kaohsiung, Taiwan; 5 Department of Public Health and Environmental Medicine, School of Medicine, College of Medicine, Kaohsiung Medical University, Kaohsiung, Taiwan; 6 Research Center for Environmental Medicine, Kaohsiung Medical University, Kaohsiung, Taiwan

**Keywords:** Biomarkers, Nephrology

## Abstract

Dyslipidemia is common in patients with chronic kidney disease (CKD), however the relationship between dyslipidemia and mortality in patients with moderate to severe CKD remains controversial. Non-high-density lipoprotein (HDL) cholesterol has been reported to be a more accurate predictor of clinical outcomes than conventional lipid measurements. Hence, the aim of this study was to investigate associations between non-HDL cholesterol and the risk of overall and cardiovascular mortality in patients with CKD stage 3–5. We enrolled 429 pre-dialysis patients with stage 3 to 5 CKD from May 2006 to January 2010. The patients were divided into four groups according to quartiles of non-HDL cholesterol. The patients were followed until death or until January 2020. During a median 11.6 years of follow-up, there were 78 (18.2%) deaths overall and 32 (7.5%) cardiovascular deaths. In adjusted models, the patients in quartile 1 (hazard ratio [HR] 3.368; 95% confidence interval [CI] 1.388–8.176; *p* = 0.007), quartile 3 (HR 3.666; 95% CI 1.486–9.044; *p* = 0.005), and quartile 4 (HR 2.868; 95% CI 1.136–7.240; *p* = 0.026) of non-HDL cholesterol had a higher risk of overall mortality (vs. quartile 2). In addition, the patients in quartile 1 (HR 19.503; 95% CI 2.185–174.0925 *p* = 0.008), quartile 3 (HR 28.702; 95% CI 2.990–275.559; *p* = 0.004), and quartile 4 (HR 11.136; 95% CI 1.126–110.108; *p* = 0.039) had a higher risk of cardiovascular mortality (vs. quartile 2). Our study showed a U-shaped relationship between non-HDL cholesterol and the risk of overall and cardiovascular mortality in patients with CKD stage 3–5. Assessing non-HDL cholesterol may help to identify subjects at high-risk of adverse outcomes.

## Introduction

Lipid disorders are a major public health issue worldwide, and many studies have reported the high rates of mortality, morbidity, and complications associated with dyslipidemia in the general population^1,2^. Dyslipidemia is common in patients with chronic kidney disease (CKD) due to severe dysregulation of key metabolic pathways and enzymes, which can lead to a decrease in high-density lipoprotein (HDL) cholesterol and increase in triglyceride-rich lipoproteins^3^. Lipid disorders in patients with CKD have been shown to both increase the risk of cardiovascular disease (CVD) and also to accelerate the progression to end-stage CKD^4–6^.

High levels of serum low-density lipoprotein (LDL) cholesterol have been clearly identified to be an important risk factor for increased CVD and mortality in the general population, however this cannot completely explain the risk associated with triglyceride-rich atherogenic lipoproteins^7^. The level of serum non-HDL cholesterol is calculated as total cholesterol minus HDL cholesterol, and it accounts for all atherogenic lipoproteins, including LDL cholesterol, lipoprotein(a), intermediate-density lipoprotein, and very LDL remnants. Atherogenic lipoproteins have been reported to be major contributors to atherosclerosis and the pathogenesis of CVD^6^. Some investigators have suggested that serum non-HDL cholesterol level may be a useful marker of the risk of atherosclerosis and CVD^7–10^. Increasing evidence also suggests that higher serum levels of non-HDL cholesterol are related to an increased risk of CVD in the general population^11–15^. In addition, non-HDL cholesterol has been reported to be a more accurate predictor of the risk and outcomes of CVD than conventional lipid measurements^12,16,17^. Some epidemiological studies have reported an association between non-HDL cholesterol and adverse CVD outcomes in patients with CKD^18–20^, whereas others have not identified such an association^21,22^. Therefore, the role of non-HDL cholesterol as a risk marker for CVD remains controversial in patients with CKD.

The influence of dyslipidemia on mortality in patients with CKD is also unclear. An association between conventional risk factors for mortality and better survival in patients with CKD, also termed “reverse epidemiology”, has been described in previous studies^23,24^. An inverse association between lipid levels and mortality in patients with CKD has also been reported in several studies^25,26^. On the other hand, a recent large randomized control trial showed that statin treatment lowered LDL cholesterol but had no substantial effect on the progression of kidney disease or overall and cause-specific mortality in patients with CKD^27^. Considering the potential link between serum lipid levels and outcomes in patients with CKD, we hypothesized that there may be a nonlinear relationship between serum concentrations of non-HDL cholesterol and mortality in patients with CKD. Therefore, the aim of the present study was to investigate the association between non-HDL cholesterol and overall and cardiovascular mortality in patients with CKD stage 3–5.

## Results

A total of 429 patients with CKD stage 3–5 were included. Their mean age was 65.9 ± 12.3 years and there were 267 men and 162 women. The patients were classified into four groups according to quartile of non-HDL cholesterol. Comparisons of the clinical characteristics of these groups are shown in Table 1. There were 107, 107, 108 and 107 patients in the four groups, respectively. Compared to the patients in quartile 1, those in quartile 4 had a higher diastolic blood pressure, and higher levels of triglycerides, total cholesterol, LDL cholesterol, and non-HDL cholesterol.

**Table 1:** Comparison of clinical characteristics according to quartiles of non-HDL cholesterol.

Characteristics	Quartile 1 (< 116.2 mg/dl) (n = 107)	Quartile 2 (116.2–143.9 mg/dl) (n = 107)	Quartile 3 (143.2–174.9 mg/dl) (n = 108)	Quartile 4 (≧ 174.9 mg/dl) (n = 107)
Age (year)	66.6 ± 12.7	66.6 ± 12.0	66.1 ± 12.5	64.1 ± 11.9
Male gender (%)	62.6	65.4	65.7	55.1
Smoking (%)	33.6	64.6	60.6	32.7
Diabetes mellitus (%)	53.3	51.4	61.1	67.3
Hypertension (%)	74.8	86.0	81.5	86.0
Coronary artery disease (%)	11.2	14.0	13.0	10.3
Congestive heart failure (%)	13.1	10.3	11.1	15.9
Underlying disease of CKD (%)				
Diabetic kidney disease	51.4	47.7	54.6	62.6
Non-diabetic glomerular disease	4.7	7.5	2.8	8.4
Hypertension	35.5	30.8	25.0	21.5
Gouty nephropathy	2.8	7.5	12.0	5.6
Others	5.6	6.5	5.6	1.9
BMI (kg/m^2^)	24.2 ± 3.9	25.2 ± 3.8	26.1 ± 4.1*	25.8 ± 4.1*
Systolic blood pressure (mmHg)	137.3 ± 22.8	139.2 ± 17.0	146.0 ± 22.9*	144.6 ± 21.9
Diastolic blood pressure (mmHg)	75.7 ± 11.2	78.2 ± 10.6	82.3 ± 14.5*	81.7 ± 14.5*
Laboratory parameters				
Fasting glucose (mg/dl)	118.9 ± 45.7	118.4 ± 43.6	130.5 ± 62.0	138.9 ± 75.6
HbA_1C_ (%)	6.5 ± 1.1	6.5 ± 1.4	7.1 ± 2.1^†^	7.3 ± 2.0*^†^
Triglyceride (mg/dl)	94 (66–128)	119 (92–166)*	167.5 (119–237.75)*^†^	200 (145–259)*^†#^
Total cholesterol (mg/dl)	145.0 ± 25.1	175.7 ± 14.9*	200.6 ± 15.5*^†^	255.6 ± 37.9*^†#^
HDL-cholesterol (mg/dl)	47.8 ± 18.0	44.9 ± 12.0	44.1 ± 13.1	44.9 ± 12.2
LDL-cholesterol (mg/dl)	73.1 ± 18.4	96.1 ± 18.0*	112.6 ± 19.4*^†^	149.7 ± 35.4*^†#^
Non-HDL cholesterol (mg/dl)	97.2 ± 16.5	130.8 ± 7.6*	156.5 ± 8.2*^†^	210.7 ± 34.5*^†#^
Hemoglobin (g/dl)	11.1 ± 2.3	11.8 ± 2.4	11.5 ± 2.5	11.6 ± 2.2
eGFR (ml/min/1.73 m^2^)	24.0 ± 14.6	27.6 ± 13.5	25.3 ± 13.7	23.7 ± 13.4
Total calcium (mg/dl)	9.3 ± 0.9	9.4 ± 0.7	9.5 ± 0.9	9.5 ± 0.8
Phosphorous (mg/dl)	4.3 ± 1.3	4.0 ± 1.0	4.1 ± 1.0	4.2 ± 0.9
Proteinuria (%)	69.2	55.7	72.9^†^	77.6^†^
Medications				
Aspirin use (%)	21.0	23.6	22.6	32.7
ACEI and/or ARB use (%)	66.7	76.4	78.3	72.1
β-Blocker use (%)	26.7	23.6	36.8	38.5*
Calcium channel blocker use (%)	42.9	54.7	59.4	63.5
Diuretics use (%)	48.6	46.2	36.8	51.9
Statin and/or fibrate use (%)	22.9	19.8	29.2	32.7
Outcome				
Overall mortality (%)	20.6	9.3	22.2	20.6
Cardiovascular mortality (%)	8.4	2.8	11.1	7.5

The study patients were stratified into 4 groups according to quartiles of non-HDL cholesterol.

*HDL* high-density lipoprotein, *CKD* chronic kidney disease, *BMI* body mass index, *LDL* low-density lipoprotein, *eGFR* estimated glomerular filtration rate, *ACEI* angiotensin converting enzyme inhibitor, *ARB* angiotensin II receptor blocker.

*p < 0.05 compared with quartile 1; ^†^*p* < 0.05 compared with quartile 2; ^#^*p* < 0.05 compared with quartile 3.

Table 2 shows unadjusted relationships among HDL cholesterol, LDL cholesterol, non-HDL cholesterol, and triglyceride quartiles and overall and cardiovascular mortality using univariate Cox proportional hazards analysis. Compared to the patients in quartile 3 of HDL cholesterol and LDL cholesterol, those in quartile 1, quartile 2, and quartile 4 were not associated with increased overall or cardiovascular mortality. However, compared to the patients in quartile 2 of non-HDL cholesterol, those in quartile 1 (hazard ratio [HR] 2.345; 95% confidence interval [CI] 1.111–4.953; *p* = 0.025), quartile 3 (HR 2.486; 95% CI 1.189–5.200; *p* = 0.016) and quartile 4 (HR 2.287; 95% CI 1.083–4.829; *p* = 0.0301) were associated with increased overall mortality. In addition, the patients in quartile 3 of non-HDL cholesterol (HR 4.160; 95% CI 1.174–14.744; *p* = 0.027) were associated with increased cardiovascular mortality. Regarding the correlation between triglyceride with outcomes, compared to the patients in quartile 2 of triglyceride, those in quartile 1 (HR 5.485; 95% CI 1.164–25.842; *p* = 0.031) were associated with increased cardiovascular mortality.

**Table 2:** Unadjusted relation of HDL-cholesterol, LDL-cholesterol, non-HDL cholesterol, and triglyceride quartiles to progression to overall and cardiovascular mortality using multivariate Cox proportional hazards model.

Unadjusted	Overall mortality		Cardiovascular mortality	
	Hazard ratio (95% CI)	*p*	Hazard ratio (95% CI)	*p*
HDL-cholesterol				
Quartile 1	1.577 (0.818–3.040)	0.174	0.924 (0.311–2.750)	0.887
Quartile 2	1.395 (0.719–2.706)	0.325	1.281 (0.477–3.439)	0.623
Quartile 3	Reference		Reference	
Quartile 4	1.336 (0.684–2.609)	0.397	1.436 (0.546–3.772)	0.463
LDL-cholesterol				
Quartile 1	1.542 (0.795–2.991)	0.200	1.262 (0.457–3.479)	0.653
Quartile 2	1.498 (0.767–2.926)	0.237	1.445 (0.538–3.881)	0.465
Quartile 3	Reference		Reference	
Quartile 4	1.587 (0.823–3.058)	0.168	1.234 (0.447–3.403)	0.685
Non-HDL cholesterol				
Quartile 1	2.345 (1.111–4.953)	0.025	3.201 (0.867–11.824)	0.081
Quartile 2	Reference		Reference	
Quartile 3	2.486 (1.189–5.200)	0.016	4.160 (1.174–14.744)	0.027
Quartile 4	2.287 (1.083–4.829)	0.030	2.772 (0.735–10.450)	0.132
Triglyceride				
Quartile 1	2.332 (0.918–5.924)	0.075	5.485 (1.164–25.842)	0.031
Quartile 2	Reference		Reference	
Quartile 3	2.161 (0.902–5.174)	0.084	2.974 (0.618–14.326)	0.174
Quartile 4	1.211 (0.477–3.075)	0.688	1.794 (0.348–9.246)	0.485

Values expressed as hazard ratio and 95% confidence interval (CI). Abbreviations are the same as in Table 1.

### Risk of overall mortality

The median follow-up period was 11.6 (10.3–12.1) years, during which 78 of the 429 patients (18.2%) died due to cardiovascular events (n = 32), malignancy (n = 6), infectious diseases (n = 34), gastrointestinal bleeding (n = 2), and others (n = 4).

### Association of non-HDL cholesterol and overall mortality

Table 3 shows the HRs of the non-HDL cholesterol quartiles for overall mortality with and without adjustments for demographic, clinical and biochemical data. Compared to the patients in quartile 2 of non-HDL cholesterol, those in quartile 1 (*p* = 0.025, *p* = 0.014, respectively), quartile 3 (*p* = 0.014, *p* = 0.013, respectively) and quartile 4 (*p* = 0.027, *p* = 0.031, respectively) were associated with increased overall mortality in the age- and sex-adjusted model and in multivariate model (1) adjusted for age, gender, smoking, diabetes mellitus, hypertension, coronary artery disease and congestive heart failure, underlying disease of CKD and body mass index (BMI). This relationship remained significant after further adjustments for systolic blood pressure ≧ 140 mmHg, fasting glucose, HbA_1C_, hemoglobin, eGFR < 30 ml/min/1.73 m^2^, total calcium, phosphorous, proteinuria, aspirin, ACEI and/or ARB, β-blocker, calcium channel blocker , diuretics and statin and/or fibrate use. The patients in quartile 1 (HR 3.368; 95% CI 1.3881–8.176; *p* = 0.007), quartile 3 (HR 3.666; 95% CI 1.486–9.044; *p* = 0.005) and quartile 4 (HR 2.868; 95% CI 1.136–7.240; *p* = 0.026) of non-HDL cholesterol were significantly associated with increased overall mortality.

**Table 3:** Relation of non-HDL cholesterol quartiles to progression to overall and cardiovascular mortality using multivariate Cox proportional hazards model.

Non-HDL cholesterol	Overall mortality		Cardiovascular mortality	
	Hazard ratio (95% CI)	*p*	Hazard ratio (95% CI)	*p*
Age and gender adjusted				
Quartile 1	2.357 (1.116–4.979)	0.025	3.231 (0.874–11.937)	0.079
Quartile 2	Reference		Reference	
Quartile 3	2.533 (1.211–5.300)	0.014	4.202 (1.185–14.899)	0.026
Quartile 4	2.337 (1.103–4.952)	0.027	2.869 (0.758–10.863)	0.121
Multivariate adjusted (1)				
Quartile 1	2.534 (1.196–5.369)	0.015	3.518 (0.948–13.065)	0.060
Quartile 2	Reference		Reference	
Quartile 3	2.546 (1.210–5.358)	0.014	4.452 (1.246–15.916)	0.022
Quartile 4	2.280 (1.070–4.859)	0.033	2.724 (0.714–10.390)	0.142
Multivariate adjusted (2)				
Quartile 1	3.368 (1.388–8.176)	0.007	19.503 (2.185–174.092)	0.008
Quartile 2	Reference		Reference	
Quartile 3	3.666 (1.486–9.044)	0.005	28.702 (2.990–275.559)	0.004
Quartile 4	2.868 (1.136–7.240)	0.026	11.136 (1.126–110.108)	0.039

Values expressed as hazard ratio and 95% confidence interval (CI). Abbreviations are the same as in Table 1.

Multivariate model 1: adjusted for age, gender, smoking, diabetes mellitus, hypertension, coronary artery disease and congestive heart failure, underlying disease of CKD and BMI.

Multivariate model 2: adjusted for age, gender, smoking, diabetes mellitus, hypertension, coronary artery disease and congestive heart failure, underlying disease of CKD, BMI, systolic blood pressure ≧ 140 mmHg, fasting glucose, HbA_1C_, hemoglobin, eGFR < 30 ml/min/1.73 m^2^, total calcium, phosphorous, proteinuria, aspirin, ACEI and/or ARB, β-blocker, calcium channel blocker, diuretics and statin and/or fibrate use.

**Figure 1:**
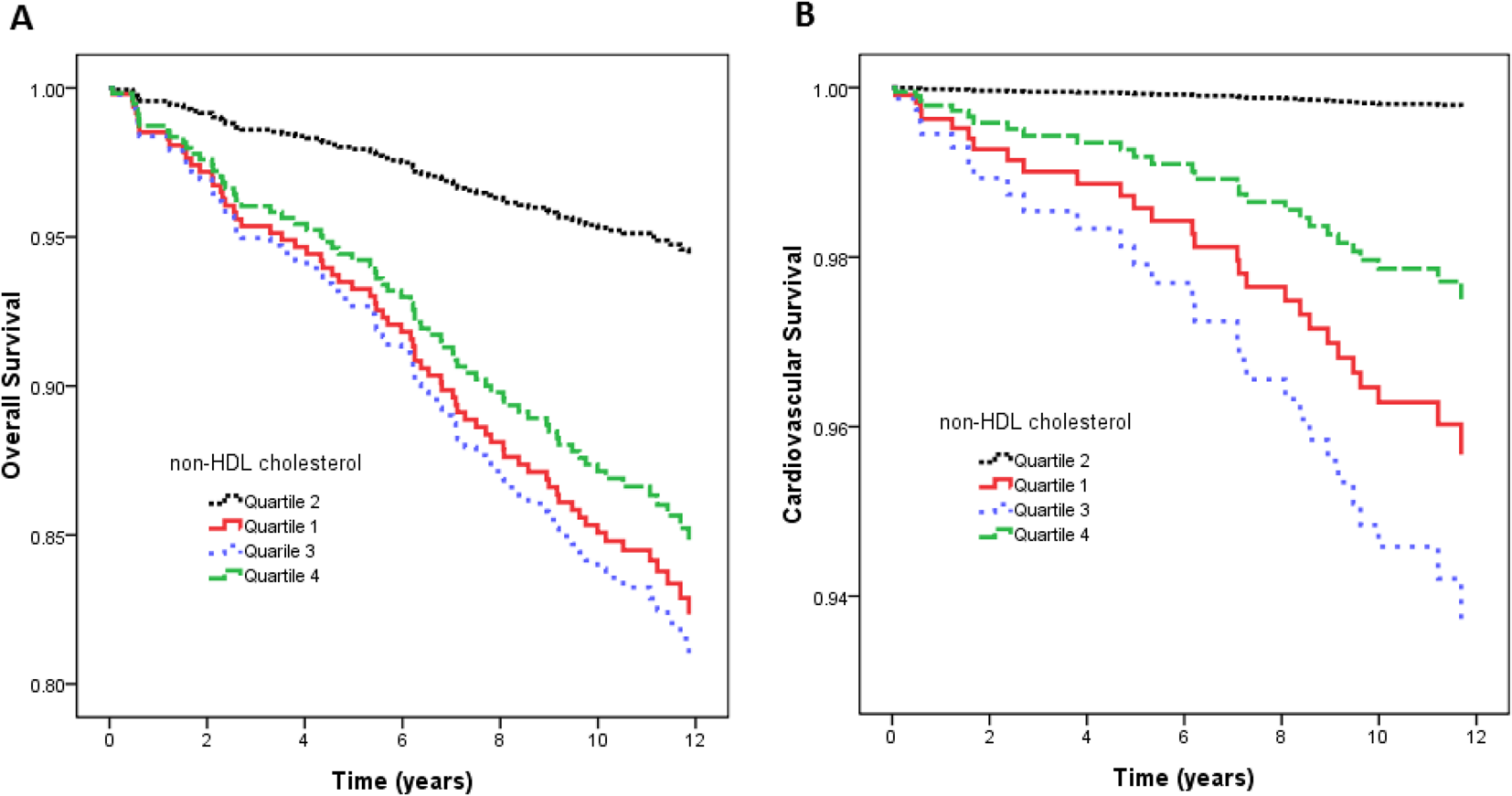
(**A**) Adjusted curves of overall survival among quartiles of non-HDL cholesterol. The group with quartile 1, quartile 3, and quartile 4 of non-HDL cholesterol had worse overall survival than that with quartile 2 of non-HDL cholesterol; (**B**) Adjusted curves of cardiovascular survival among quartiles of non-HDL cholesterol. The group with quartile 1, quartile 3, and quartile 4 of non-HDL cholesterol had worse cardiovascular survival than that with quartile 2 of non-HDL cholesterol.

**Figure 2:**
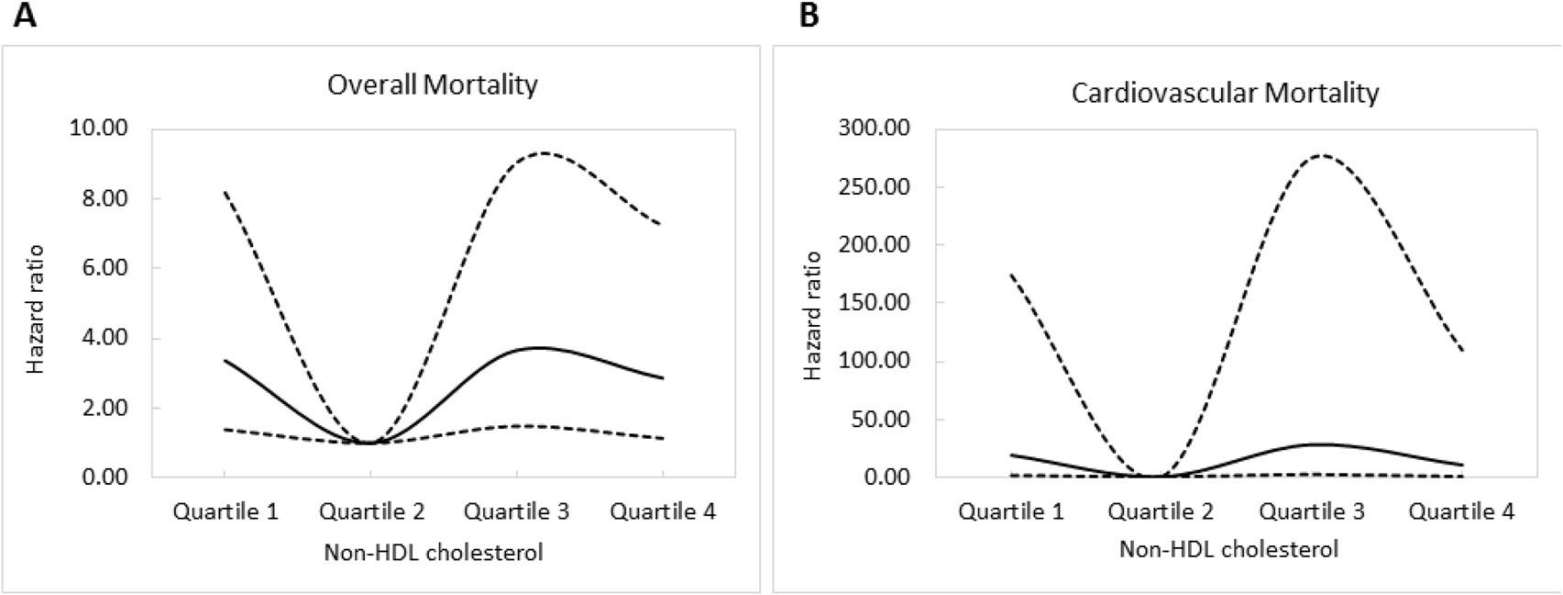
Associations of non-HDL cholesterol with overall (**A**) and cardiovascular (**B**) mortality in CKD examined using a Cox proportional hazards model after adjusting for demographics, comorbid conditions, laboratory data, and use of medications.

Figure 1A illustrates the adjusted curves of overall survival among the quartiles of non-HDL cholesterol. The patients in quartile 1, quartile 3 and quartile 4 of non-HDL cholesterol had worse overall survival than those in quartile 2. Figure 2A illustrates the associations between non-HDL cholesterol and overall mortality using a Cox proportional hazards model after adjusting for demographic data, comorbid conditions, laboratory data, and the use of medications.

### Association of triglyceride and overall mortality

Table 4 shows the HRs of the triglyceride quartiles for overall mortality with and without adjustments for demographic, clinical and biochemical data. Compared to the patients in quartile 2 of triglyceride, the patients in quartile 3 (HR 2.392; 95% CI 1.121–5.107; *p* = 0.024) of triglyceride were significantly associated with increased overall mortality after multivariate adjustments.

**Table 4:** Relation of triglyceride quartiles to progression to overall and cardiovascular mortality using multivariate Cox proportional hazards model.

Non-HDL cholesterol	Overall mortality		Cardiovascular mortality	
	Hazard ratio (95% CI)	*p*	Hazard ratio (95% CI)	*p*
Age and gender adjusted				
Quartile 1	1.612 (0.809–3.210)	0.175	2.717 (0.846–8.726)	0.093
Quartile 2	Reference		Reference	
Quartile 3	1.880 (0.971–3.640)	0.061	2.432 (0.747–7.918)	0.140
Quartile 4	1.480 (0.747–2.933)	0.262	2.319 (0.713–7.542)	0.162
Multivariate adjusted (1)				
Quartile 1	1.835 (0.911–3.696)	0.089	3.180 (0.966–10.470)	0.057
Quartile 2	Reference		Reference	
Quartile 3	1.874 (0.950–3.697)	0.070	2.465 (0.736–8.263)	0.144
Quartile 4	1.501 (0.741–3.039)	0.259	2.544 (0.758–8.538)	0.131
Multivariate adjusted (2)				
Quartile 1	2.081 (0.968–4.475)	0.061	5.545 (1.319–23.315)	0.019
Quartile 2	Reference		Reference	
Quartile 3	2.392 (1.121–5.107)	0.024	5.453 (1.169–25.430)	0.031
Quartile 4	1.599 (0.706–3.623)	0.261	3.840 (0.793–18.597)	0.095

Values expressed as hazard ratio and 95% confidence interval (CI). Abbreviations are the same as in Table 1.

Multivariate model 1: adjusted for age, gender, smoking, diabetes mellitus, hypertension, coronary artery disease and congestive heart failure, underlying disease of CKD and BMI.

Multivariate model 2: adjusted for age, gender, smoking, diabetes mellitus, hypertension, coronary artery disease and congestive heart failure, underlying disease of CKD, BMI, systolic blood pressure ≧ 140 mmHg, fasting glucose, HbA_1C_, hemoglobin, eGFR < 30 ml/min/1.73 m^2^, total calcium, phosphorous, proteinuria, aspirin, ACEI and/or ARB, β-blocker, calcium channel blocker , diuretics and statin and/or fibrate use.

**Figure 3:**
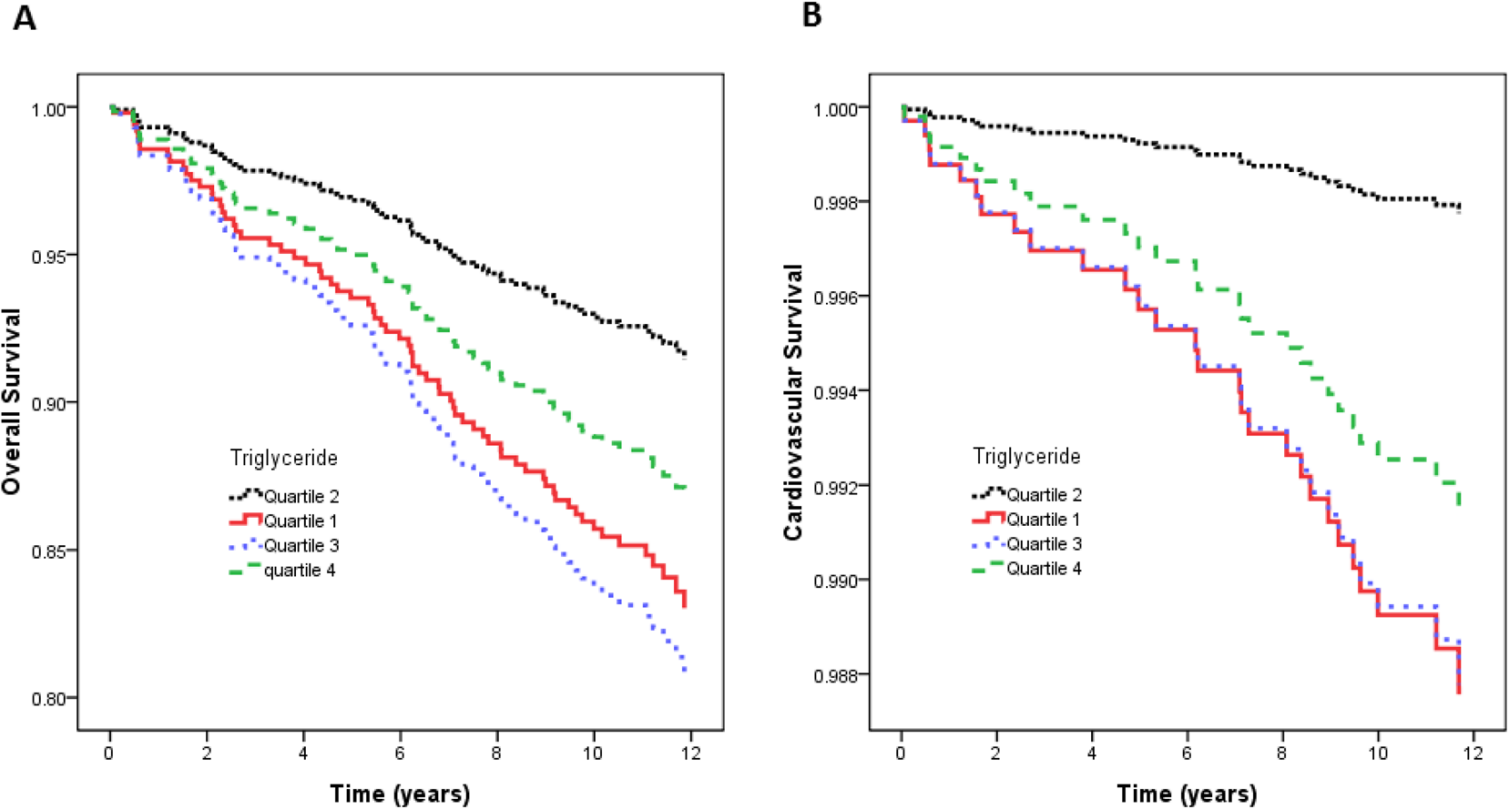
(**A**) Adjusted curves of overall survival among quartiles of triglyceride. The group with quartile 3 of triglyceride had worse overall survival than that with quartile 2 of triglyceride; (**B**) Adjusted curves of cardiovascular survival among quartiles of triglyceride. The group with quartile 1, and quartile 3 of triglyceride had worse cardiovascular survival than that with quartile 2 of triglyceride.

**Figure 4:**
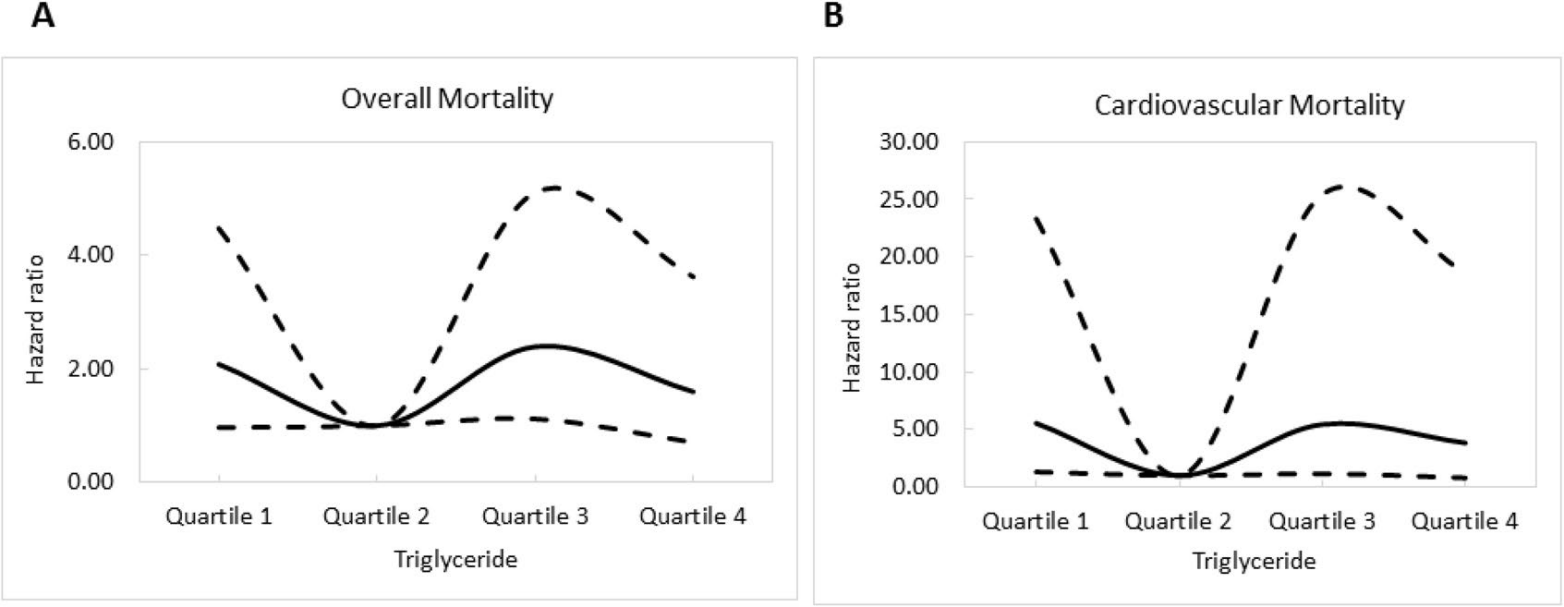
Associations of triglyceride with overall (**A**) and cardiovascular (**B**) mortality in CKD examined using a Cox proportional hazards model after adjusting for demographics, comorbid conditions, laboratory data, and use of medications.

Figure 3A illustrates the adjusted curves of overall survival among the quartiles of triglyceride. The patients in quartile 3 of triglyceride had worse overall survival than those in quartile 2. Figure 4A illustrates the associations between triglyceride and overall mortality using a Cox proportional hazards model after multivariate adjustments.

### Risk of cardiovascular mortality

Of the 32 cardiovascular deaths recorded during follow-up, 18 were due to heart failure, three due to myocardial infarction, and 11 due to ventricular fibrillation.

### Association of non-HDL cholesterol and cardiovascular mortality

Multivariate Cox proportional hazards regression analysis of the four study groups for cardiovascular mortality is shown in Table 3. Compared to the patients in quartile 2 of non-HDL cholesterol, those in quartile 3 (*p* = 0.026, *p* = 0.022, respectively) were associated with increased cardiovascular mortality in the age- and sex-adjusted model and in multivariate model (1), but those in quartile 1 (*p* = 0.079, *p* = 0.060, respectively) and quartile 4 (*p* = 0.121, *p* = 0.142, respectively) were not. After further adjustments in multivariate model (2), the patients in quartile 1 (HR 19.503; 95% CI 2.185–174.092; *p* = 0.008), quartile 3 (HR 28.702; 95% CI 2.990 to 275.559; *p* = 0.004), and quartile 4 (HR 11.136; 95% CI 1.126–110.108; *p* = 0.039) of non-HDL cholesterol were significantly associated with increased cardiovascular mortality.

Figure 1B illustrates adjusted curves of cardiovascular survival among the quartiles of non-HDL cholesterol. The patients in quartile 1, quartile 3, and quartile 4 of non-HDL cholesterol had worse cardiovascular survival than those in quartile 2. Figure 2B illustrates the associations between non-HDL cholesterol and cardiovascular mortality using a Cox proportional hazards model after multiple adjustments.

### Association of triglyceride and cardiovascular mortality

Table 4 shows the HRs of the triglyceride quartiles for cardiovascular mortality with and without adjustments for demographic, clinical and biochemical data. Compared to the patients in quartile 2 of triglyceride, the patients in quartile 1 (HR 5.545; 95% CI 1.319–23.315; *p* = 0.019), and quartile 3 (HR 5.453; 95% CI 1.169–25.430; *p* = 0.031) of triglyceride were significantly associated with increased cardiovascular mortality after multivariate adjustments.

Figure 3B illustrates the adjusted curves of overall survival among the quartiles of triglyceride. The patients in quartile 1 and quartile 3 of triglyceride had worse cardiovascular survival than those in quartile 2. Figure 4B illustrates the associations between triglyceride and cardiovascular mortality using a Cox proportional hazards model after multivariate adjustments.

## Discussion

In this study, we evaluated the association between non-HDL cholesterol and mortality in patients with CKD stages 3–5. Our results showed that both a lower and higher quartile of non-HDL cholesterol were significantly associated with both overall and cardiovascular mortality in stage 3–5 CKD patients. In addition, non-HDL cholesterol appeared to be a stronger predictor of future mortality than LDL cholesterol, HDL cholesterol in these patients.

There are several potential advantages to using non-HDL cholesterol as an index of lipid abnormality in patients with CKD. First, it provides an assessment of all atherogenic lipoproteins including LDL, intermediate-density lipoprotein, very LDL, and lipoprotein(a). Patients with CKD have a different serum lipid profile pattern to the general population. Dyslipidemia in patients with CKD is characterized by hypertriglyceridemia, increases in triglyceride-rich remnant lipoproteins and lipoprotein(a), and a reduction in HDL cholesterol^28^. Accordingly, the contribution of other lipoproteins may be missed if only one lipoprotein level is used. We also found a similar pattern of a nonlinear relationship with mortality when using LDL cholesterol and HDL cholesterol as the exposure variables, however the results were not statistically significant. Therefore, serum non-HDL cholesterol level may be a more comprehensive marker of dyslipidemia in patients with CKD. Second, non-HDL cholesterol can easily be calculated from total cholesterol and HDL cholesterol, which are readily available from a standard lipid profile. Moreover, total cholesterol and HDL cholesterol can be measured accurately even in a non-fasting state as opposed to LDL cholesterol. Third, non-HDL cholesterol has been reported in several studies to be superior to LDL cholesterol in predicting CVD risk^12,16,17^. In addition, many studies have also reported strong positive associations between serum levels of non-HDL cholesterol and adverse cardiovascular outcomes in patients with CKD^18–20^.

One important finding of our study is that the CKD patients with higher serum non-HDL cholesterol levels had an increased risk of mortality, which is similar to the general population. There are several potential explanations for the underlying mechanisms of this relationship. First, non-HDL cholesterol includes all potentially atherogenic lipids, and it has been shown to be a good surrogate marker of triglycerides and their remnants^29^. Higher risks of fatty streaks, vascular stenosis, carotid intima-media thickness and angiographic progression of coronary artery disease have also been reported to be positively associated with serum non-HDL cholesterol levels^30^. Second, levels of non-HDL cholesterol have been reported to be well correlated with levels of apolipoprotein B (ApoB), the major protein on pro-atherogenic lipoproteins^31^. Every pro-atherogenic lipoprotein, including very LDL, very LDL remnants, LDL and lipoprotein(a) particles, contain one molecule of ApoB. Experimental data have shown that the atherogenicity of lipoproteins containing ApoB is associated with subendothelial retention, arterial wall proteoglycans, and proinflammatory properties^32^. Third, an inverse correlation between non-HDL cholesterol and LDL particle size has been reported^33^, with small dense LDL particles being more prone to oxidization and consequently being more atherogenic. In addition, prospective studies have reported associations between the preferential accumulation of small dense LDL particles and an increased risk of cardiovascular events^34–36^. Moreover, individuals with a preferential accumulation of small dense LDL particles have been shown to have higher levels of triglycerides and total cholesterol and a lower level of HDL cholesterol^37^, which are all features of dyslipidemia in patients with CKD.

Another important finding of this study is that the patients with lower serum non-HDL cholesterol levels also had a poor prognosis. Non-HDL cholesterol levels are calculated by subtracting HDL cholesterol levels from total cholesterol levels, and a low non-HDL cholesterol level could represent a low total cholesterol or high HDL cholesterol level. Several observational studies have reported an association between the risk of mortality and a lower total cholesterol level among patients with CKD, possibly due to the high prevalence of malnutrition and inflammation^38,39^. Malnutrition can lead to worse outcomes by worsening existing inflammation and hastening the progression of atherosclerosis^40–42^. On the other hand, a recent study demonstrated a U-shaped association between mortality and HDL cholesterol both in patients with and without kidney disease^43^. In addition, a large cohort study of 38,377 patients with an eGFR 15–59 ml/min/1.73 m^2^ reported that a serum HDL cholesterol level > 60 mg/dl was associated with higher risks of all-cause and cardiovascular mortality in male CKD patients^44^. Although low HDL cholesterol is a typical feature of lipid disturbance in CKD, many reports have demonstrated a decrease in HDL antioxidants and anti-inflammatory activity in patients with CKD at any stage^45–47^. Furthermore, many studies have demonstrated that HDL cholesterol can change from an anti-inflammatory to a pro-inflammatory molecule in patients with advanced CKD^48,49^. It is therefore plausible that HDL cholesterol in such patients can paradoxically increase their cardiovascular risk leading to a worse outcome regardless of the serum concentration of HDL cholesterol. The combination of these two factors may explain the findings of the present study.

The last important finding of this study is that the patients with both lower and higher serum triglyceride levels also had an increased risk of overall and cardiovascular mortality among CKD stage 3–5 patients. The relationship between triglyceride and mortality or cardiovascular outcomes in CKD population was inconsistent in previous studies. Kovesdy et al.^25^ reported the association of lower triglyceride levels with higher overall and cardiovascular mortality in pre-dialysis patients. Nonetheless, the inverse association between triglycerides and mortality was weakened after adjustment for malnutrition-inflammation-cachexia syndrome. Shlipak et al.^50^ also demonstrated similar findings in a community-based cohort of elderly persons with CKD. Moreover, in a cohort of nondiabetic stage 3 to 4 CKD subjects, Chawla et al.^22^ suggested that hyperlipidemia did not appear to be independently associated with higher risk for overall and cardiovascular mortality. On the other hand, among a large cohort of U.S. veterans, Soohoo et al.^51^ showed a U-shaped association between triglycerides and all-cause mortality and cardiovascular outcome in patients with CKD stage 3 to 4, although there were no statistics significance in subjects with CKD stage 5. In the present study, both lower and higher triglyceride levels were associated with poor prognosis in patients with CKD stage 3 to 5.

Although LDL cholesterol is the main therapeutic target for patients with CKD, evaluating complete lipid profiles including total cholesterol, LDL cholesterol, HDL cholesterol, triglycerides, and non-HDL cholesterol is recommended by the current Kidney Disease: Improving Global Outcomes (KDIGO) clinical practice guidelines^52^. The current KDIGO guidelines also recommend the use of statins for all patients aged > 50 years who are not receiving chronic dialysis or have undergone a kidney transplantation regardless of LDL cholesterol level. Current evidence suggests against the use of LDL cholesterol to identify CKD patients who should receive cholesterol-lowering treatment due to the inconclusive association between dyslipidemia and mortality in patients with CKD. In the Study of Heart and Renal Protection (SHARP) trial^27^, statin therapy targeting serum LDL cholesterol levels in CKD patients was not shown to improve cardiovascular or overall survival. In addition, in the present study, we demonstrated a U-shaped relationship between mortality and non-HDL cholesterol. Our results showed that the patients with very low levels of serum non-HDL cholesterol, which is recommended as ideal or optimal by current guidelines, also had a poor prognosis. Therefore, the decision to initiate statin treatment in patients with CKD should focus on the underlying cardiovascular risk and malnutrition-inflammation status, not just the lipid profile.

There are several limitations to this study. First, the sample size and number of events were small, and the study patients were included from one regional hospital in southern Taiwan. Thus, the generalizability of our results may be limited. Second, we evaluated baseline serum non-HDL cholesterol levels only once. The subsequent use of lipid-modifying medication could have altered the lipid levels in some subjects and may have led to some misclassifications. However, such measurements are supported by the current KDIGO clinical practice guidelines, which recommend evaluating the lipid profile only at the initial presentation^52^. Third, we lacked details of lipoprotein fractions (including apolipoprotein A and ApoB levels) to explain potential mechanistic pathways responsible for the observed associations. Lastly, some important variables influencing CKD outcomes (e.g. alcohol consumption habit, heart rate, arrhythmia, urine protein-to-creatinine or albumin to creatinine ratio, sodium, potassium, hematuria or renal ultrasonography) were lacking.

This study of patients with CKD stage 3–5 showed a U-shaped relationship between quartiles of serum non-HDL cholesterol and the risk of cardiovascular and overall mortality. Serum non-HDL cholesterol levels may be a more accurate marker of outcomes than conventional lipid measurements. Further studies are needed to investigate the potential value of using non-HDL cholesterol as a primary target for lipid management in patients with CKD.

## Study patients and methods

### Study patients and design

This study was conducted in a regional hospital in southern Taiwan. We consecutively enrolled 505 pre-dialysis patients with CKD stage 3 to 5 according to the National Kidney Foundation-Kidney Disease Outcomes Quality Initiative (K/DOQI) guidelines^53^ from our Outpatient Department of Internal Medicine from May 2006 to January 2010. All of the patients had evidence of kidney damage lasting for at least 3 months. The stages of CKD were defined according to estimated glomerular filtration rate (eGFR) as follows: stage 3, 30 to 59 ml/min/1.73 m^2^; stage 4, 15 to 29 ml/min/1.73 m^2^; and stage 5, < 15 ml/min/1.73 m^2^. Seventy-six patients without complete lipid measurements during the enrollment period were excluded, and the remaining 429 patients (mean age 65.9 ± 12.3 years, 267 males) were included in this study. The study protocol was approved by the Institutional Review Board of Kaohsiung Medical University Hospital, and all of the patients provided written informed consent to participate in this study. All methods were carried out in accordance with the approved guidelines.

### Collection of demographic, medical and laboratory data

Baseline variables including demographic features (age and sex), smoking history, medical history (diabetes mellitus, hypertension, coronary artery disease and congestive heart failure), systolic and diastolic blood pressure, underlying disease of CKD, BMI, laboratory data (fasting glucose, HbA_1C_, hemoglobin, triglycerides, total cholesterol, LDL cholesterol, HDL cholesterol, non-HDL cholesterol, total calcium, phosphate and proteinuria), and medication history (aspirin, ACEI and/or ARB, β-blocker, calcium channel blocker, diuretics, statins and fibrates). The demographic features were obtained at baseline, and medical histories were obtained by chart review. Fasting blood samples and urine samples were obtained within 1 month of enrollment, and laboratory tests were performed on an autoanalyzer (Roche Diagnostics GmbH, D-68298 Mannheim COBAS Integra 400). Serum creatinine levels were evaluated using the compensated Jaffé (kinetic alkaline picrate) method on a Roche/Integra 400 Analyzer (Roche Diagnostics, Mannheim, Germany) using a calibrator traceable to isotope-dilution mass spectrometry^54^. The eGFR was calculated using the 4-variable Modification of Diet in Renal Disease (MDRD) equation^55^. Dipstick tests were used to assess proteinuria (Hema-Combistix; Bayer Diagnostics, Dublin, Ireland), with a test result of ≥ 1 + being defined as positive.

### Definition of overall and cardiovascular mortality

Two cardiologists confirmed and ascertained cases of overall and cardiovascular death from medical records, and disagreements were resolved through consensus with a third cardiologist. Patients were followed until death or until January 2020.

### Statistical analysis

Statistical analysis was performed using SPSS version 19.0 for Windows (SPSS Inc. Chicago, USA). Data were expressed as percentage, mean ± standard deviation, or median (25th–75th percentile) for triglycerides. Among-group comparisons were performed using one-way analysis of variance followed by a Bonferroni post hoc test. Cox proportional hazards analysis was used to investigate relationships between quartiles of lipid profiles with overall and cardiovascular mortality. Quartile 3 of HDL-cholesterol and LDL-cholesterol and quartile 2 of triglyceride non-HDL cholesterol were used as reference categories according to the lowest incidence rate. Associations between quartiles of non-HDL cholesterol and triglyceride between overall and cardiovascular mortality were assessed using three models as follows: (1) age and sex; (2) clinical risk factors were added; and (3) biochemical factors were added. Adjusted survival curves for overall and cardiovascular survival were illustrated using Cox regression analysis. A difference was considered to be significant at *p* < 0.05.

## References

[1] Eaton CB (2005). Hyperlipidemia. Prim. Care.

[2] Qi L (2015). Prevalence and risk factors associated with dyslipidemia in Chongqing, China. Int. J. Environ. Res. Public Health.

[3] Vaziri ND, Norris K (2011). Lipid disorders and their relevance to outcomes in chronic kidney disease. Blood Purif..

[4] Go AS, Chertow GM, Fan D, McCulloch CE, Hsu C-Y (2004). Chronic kidney disease and the risks of death, cardiovascular events, and hospitalization. N. Engl. J. Med..

[5] Trevisan R, Dodesini AR, Lepore G (2006). Lipids and renal disease. J. Am. Soc. Nephrol..

[6] Panel, E. D. & Grundy, S. M (2013). An International Atherosclerosis Society Position Paper: global recommendations for the management of dyslipidemia. J. Clin. Lipidol..

[7] Expert Panel on Detection E (2001). Executive summary of the third report of the National Cholesterol Education Program (NCEP) expert panel on detection, evaluation, and treatment of high blood cholesterol in adults (Adult Treatment Panel III). JAMA.

[8] Packard CJ, Saito Y (2004). Non-HDL cholesterol as a measure of atherosclerotic risk. J. Atheroscler. Thromb..

[9] Blaha MJ, Blumenthal RS, Brinton EA, Jacobson TA, National Lipid Association Taskforce on Non-HDL Cholesterol (2008). The importance of non-HDL cholesterol reporting in lipid management. J. Clin. Lipidol..

[10] Verbeek R, Hovingh GK, Boekholdt SM (2015). Non-high-density lipoprotein cholesterol: current status as cardiovascular marker. Curr. Opin. Lipidol..

[11] Cui Y (2001). Non-high-density lipoprotein cholesterol level as a predictor of cardiovascular disease mortality. Arch. Intern. Med..

[12] Ridker PM, Rifai N, Cook NR, Bradwin G, Buring JE (2005). Non-HDL cholesterol, apolipoproteins AI and B100, standard lipid measures, lipid ratios, and CRP as risk factors for cardiovascular disease in women. JAMA.

[13] Arsenault BJ (2009). Beyond low-density lipoprotein cholesterol: respective contributions of non-high-density lipoprotein cholesterol levels, triglycerides, and the total cholesterol/high-density lipoprotein cholesterol ratio to coronary heart disease risk in apparently healthy men and women. J. Am. Coll. Cardiol..

[14] Tanabe N (2010). Serum total and non-high-density lipoprotein cholesterol and the risk prediction of cardiovascular events. Circ. J..

[15] Holewijn S, Den Heijer M, Swinkels DW, Stalenhoef A, De Graaf J (2010). Apolipoprotein B, non-HDL cholesterol and LDL cholesterol for identifying individuals at increased cardiovascular risk. J. Intern. Med..

[16] Miller M, Ginsberg HN, Schaefer EJ (2008). Relative atherogenicity and predictive value of non-high-density lipoprotein cholesterol for coronary heart disease. Am. J. Cardiol..

[17] van Deventer HE (2011). Non-HDL cholesterol shows improved accuracy for cardiovascular risk score classification compared to direct or calculated LDL cholesterol in a dyslipidemic population. Clin. Chem..

[18] Shoji T, Masakane I, Watanabe Y, Iseki K, Tsubakihara Y (2011). Elevated non-high-density lipoprotein cholesterol (non-HDL-C) predicts atherosclerotic cardiovascular events in hemodialysis patients. Clin. J. Am. Soc. Nephrol..

[19] Holzmann MJ (2012). Dyslipidemia is a strong predictor of myocardial infarction in subjects with chronic kidney disease. Ann. Med..

[20] Usui T (2017). Serum non-high-density lipoprotein cholesterol and risk of cardiovascular disease in community dwellers with chronic kidney disease: the Hisayama study. J. Atheroscler. Thromb..

[21] Schupf N (2005). Relationship between plasma lipids and all-cause mortality in nondemented elderly. J. Am. Geriatr. Soc..

[22] Chawla V (2010). Hyperlipidemia and long-term outcomes in nondiabetic chronic kidney disease. Clin. J. Am. Soc. Nephrol..

[23] Kalantar-Zadeh K, Block G, Humphreys MH, Kopple JD (2003). Reverse epidemiology of cardiovascular risk factors in maintenance dialysis patients. Kidney Int..

[24] Kovesdy CP, Anderson JE (2007). Cardiovascular and survival paradoxes in dialysis patients: reverse epidemiology in patients with chronic kidney disease who are not yet on dialysis. Semin. Dial..

[25] Kovesdy CP, Anderson JE, Kalantar-Zadeh K (2007). Inverse association between lipid levels and mortality in men with chronic kidney disease who are not yet on dialysis: effects of case mix and the malnutrition-inflammation-cachexia syndrome. J. Am. Soc. Nephrol..

[26] Chang TI (2018). Inverse association between serum non-high-density lipoprotein cholesterol levels and mortality in patients undergoing incident hemodialysis. J. Am. Heart Assoc..

[27] Baigent C (2011). The effects of lowering LDL cholesterol with simvastatin plus ezetimibe in patients with chronic kidney disease (Study of Heart and Renal Protection): a randomised placebo-controlled trial. The Lancet.

[28] Kwan BC, Kronenberg F, Beddhu S, Cheung AK (2007). Lipoprotein metabolism and lipid management in chronic kidney disease. J. Am. Soc. Nephrol..

[29] Assessment R (2009). Major lipids, apolipoproteins, and risk of vascular disease. JAMA.

[30] Bittner V (2004). Non-high-density lipoprotein cholesterol: an alternate target for lipid-lowering therapy. Prev. Cardiol..

[31] Leroux G (2000). Influence of triglyceride concentration on the relationship between lipoprotein cholesterol and apolipoprotein B and AI levels. Metabolism.

[32] Shapiro MD, Fazio S (2017). Apolipoprotein B-containing lipoproteins and atherosclerotic cardiovascular disease. F1000Res.

[33] El Harchaoui K (2007). Value of low-density lipoprotein particle number and size as predictors of coronary artery disease in apparently healthy men and women: the EPIC-Norfolk Prospective Population Study. J. Am. Coll. Cardiol..

[34] Arai H (2013). Small dense low-density lipoproteins cholesterol can predict incident cardiovascular disease in an urban Japanese cohort: the Suita study. J. Atheroscler. Thromb..

[35] Williams PT, Zhao X-Q, Marcovina SM, Brown BG, Krauss RM (2013). Levels of cholesterol in small LDL particles predict atherosclerosis progression and incident CHD in the HDL-Atherosclerosis Treatment Study (HATS). PLoS ONE.

[36] Hoogeveen RC (2014). Small dense low-density lipoprotein-cholesterol concentrations predict risk for coronary heart disease: the Atherosclerosis Risk in Communities (ARIC) study. Arterioscler. Thromb. Vasc. Biol..

[37] St-Pierre AC (2005). Low-density lipoprotein subfractions and the long-term risk of ischemic heart disease in men: 13-year follow-up data from the Quebec Cardiovascular Study. Arterioscler. Thromb. Vasc. Biol..

[38] Liu Y (2004). Association between cholesterol level and mortality in dialysis patients: role of inflammation and malnutrition. JAMA.

[39] Contreras G (2010). Malnutrition-inflammation modifies the relationship of cholesterol with cardiovascular disease. J. Am. Soc. Nephrol..

[40] Levin NW, Handelman GJ, Coresh J, Port FK, Kaysen GA (2007). Reverse epidemiology: a confusing, confounding, and inaccurate term. Semin. Dial..

[41] Chen S-C (2011). Impaired left ventricular systolic function and increased brachial-ankle pulse-wave velocity are independently associated with rapid renal function progression. Hypertens. Res..

[42] Chen S-C (2011). Echocardiographic parameters are independently associated with rate of renal function decline and progression to dialysis in patients with chronic kidney disease. Clin. J. Am. Soc. Nephrol..

[43] Bowe B (2016). High density lipoprotein cholesterol and the risk of all-cause mortality among US veterans. Clin. J. Am. Soc. Nephrol..

[44] Navaneethan SD (2018). High-density lipoprotein cholesterol and causes of death in chronic kidney disease. J. Clin. Lipidol..

[45] Moradi H, Pahl MV, Elahimehr R, Vaziri ND (2009). Impaired antioxidant activity of high-density lipoprotein in chronic kidney disease. Transl. Res..

[46] Vaziri ND, Navab M, Fogelman AM (2010). HDL metabolism and activity in chronic kidney disease. Nat. Rev. Nephrol..

[47] Gluba-Brzozka A, Franczyk B, Rysz J (2019). Cholesterol disturbances and the role of proper nutrition in CKD patients. Nutrients.

[48] Yamamoto S (2012). Dysfunctional high-density lipoprotein in patients on chronic hemodialysis. J. Am. Coll. Cardiol..

[49] Moradi H, Vaziri ND, Kashyap ML, Said HM, Kalantar-Zadeh K (2013). Role of HDL dysfunction in end-stage renal disease: a double-edged sword. J. Ren. Nutr..

[50] Shlipak MG (2005). Cardiovascular mortality risk in chronic kidney disease: comparison of traditional and novel risk factors. JAMA.

[51] Soohoo M (2019). Serum triglycerides and mortality risk across stages of chronic kidney disease in 2 million US veterans. J. Clin. Lipidol..

[52] Wanner C, Tonelli M (2014). KDIGO Clinical Practice Guideline for Lipid Management in CKD: summary of recommendation statements and clinical approach to the patient. Kidney Int..

[53] Levey AS (2002). K/DOQI clinical practice guidelines for chronic kidney disease: evaluation, classification, and stratification. Am. J. Kidney Dis..

[54] Vickery S, Stevens PE, Dalton RN, van Lente F, Lamb EJ (2006). Does the ID-MS traceable MDRD equation work and is it suitable for use with compensated Jaffe and enzymatic creatinine assays?. Nephrol. Dial. Transplant..

[55] Levey AS (1999). A more accurate method to estimate glomerular filtration rate from serum creatinine: a new prediction equation. Ann. Intern. Med..

